# Unveiling heterocyclic aromatic amines (HAAs) in thermally processed meat products: Formation, toxicity, and strategies for reduction – A comprehensive review

**DOI:** 10.1016/j.fochx.2023.100833

**Published:** 2023-08-09

**Authors:** Haijie Wang, Xiaoran Chu, Pengfei Du, Hongjun He, Feng He, Yaobo Liu, Weiting Wang, Yanli Ma, Lei Wen, Yuanshang Wang, Fatih Oz, A.M. Abd El-Aty

**Affiliations:** a Institute of Food & Nutrition Science and Technology, Shandong Academy of Agricultural Sciences, Shandong Provincial Key Laboratory of Agro-Products Processing Technology, Key Laboratory of Novel Food Resources Processing, Ministry of Agriculture, Jinan 250100, China; b College of Life Sciences, Yantai University, Yantai 264005, China; c School of Life Sciences and Food Engineering, Hebei University of Engineering, Handan 056038, China; d College of Food Science and Technology, Huazhong Agricultural University, Wuhan 430070, China; e Department of Food Engineering, Faculty of Agriculture, Ataturk University, Erzurum 25240, Turkey; f Department of Pharmacology, Faculty of Veterinary Medicine, Cairo University, 12211 Giza, Egypt; g Department of Medical Pharmacology, Medical Faculty, Ataturk University, 25240 Erzurum, Turkey

**Keywords:** Heterocyclic aromatic amines, Protein-rich foods, Inhibition mechanism, Formation mechanism, Toxicity, Distribution, Carcinogen, 2-Amino-3-methyl-imidazole[4,5-f]quinoline (PubChem CID: 53462), 2-Amino-3,4-dimethylimidazo[4,5-f]quinoline (PubChem CID: 62274), 3-Methyl-3H-imidazo[4,5-f]quinoxalin-2-amine (PubChem CID: 105041), 2-Amino-3,8-dimethylimidazo[4,5-f]quinoxaline (PubChem CID: 62275), 2-Amino-1-methyl-6-phenylimidazo[4,5-b]pyridine (PubChem CID: 1530), Harman (PubChem CID: 5281404), Norharman (PubChem CID: 64961)

## Abstract

This comprehensive review focuses on heterocyclic aromatic amines (HAAs), a class of chemicals that commonly form during the cooking or processing of protein-rich foods. The International Agency for Research on Cancer (IARC) has categorized certain HAAs as probable human carcinogens, highlighting the significance of studying their formation and control in food safety research. The main objective of this review is to address the knowledge gaps regarding HAAs formation and propose approaches to reduce their potential toxicity during thermal processing. By summarizing the mechanisms involved in HAAs formation and inhibition, the review encompasses both conventional and recent detection methods. Furthermore, it explores the distribution of HAAs in thermally processed meats prepared through various cooking techniques and examines their relative toxicity. Additionally, considering that the Maillard reaction, responsible for HAAs formation, also contributes to the unique flavors and aromas of cooked meat products, this review investigates the potential effects of inhibiting HAAs formation on flavor substances. A thorough understanding of these complex interactions provides a foundation for developing targeted interventions to minimize the formation of HAAs and other harmful compounds during food processing.

## Highlights

• A comprehensive summary of the inhibition mechanism of HAA formation is provided.
• The interactions that lead to the formation of various hazardous substances are highlighted.
• It explores the effects of inhibiting HAAs formation on flavor substances.
• The rapid advancements in HAAs detection technology are discussed.
• The distribution of HAAs in different thermally processed meats and their relative toxicity is illustrated.

## Graphical abstract

**Figure:**
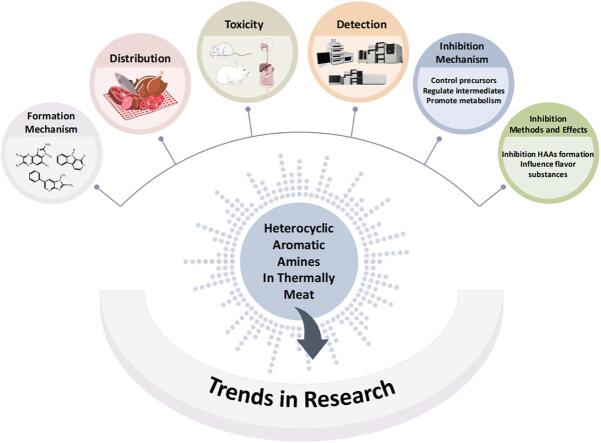

## 1 Introduction

**Fig. 1:**
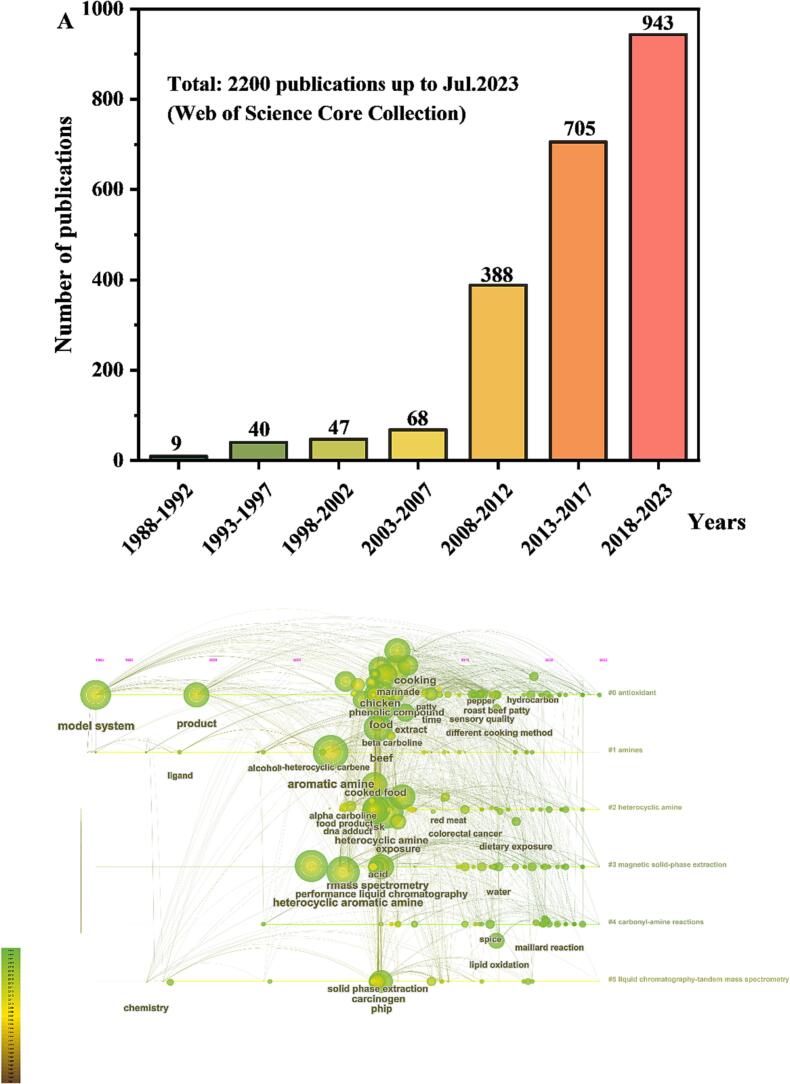
Publication trends of papers related to HAAs. (A) Number of HAA-related papers published over the past 35 years. (B) Timeline map depicting the research progress and presence of HAA-related keywords.

Heterocyclic aromatic amines (HAAs) are a diverse group of harmful substances characterized by their heterocyclic structures, which are produced during the thermal processing of protein-rich food sources, particularly meat (Gibis 2016). Extensive research using animal models, such as rats and mice, has demonstrated that these substances have carcinogenic and mutagenic effects (Im, Choi, and Kim 2010). The escalating academic interest in HAAs is reflected in the rising number of publications worldwide, as illustrated in Fig. 1A. Moreover, another chart highlights the recent research emphasis on the formation and inhibition of HAAs in thermally processed meat (Fig. 1B).

**Fig. 2:**
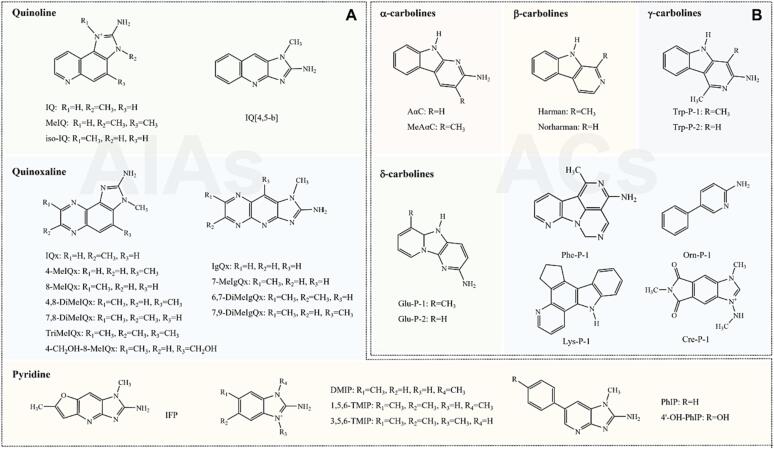
Structural illustrations of HAAs. (A) Diagrams showing the structures of AIAs. (B) Diagrams depicting the structures of ACs.

The Ames/*Salmonella* assay has identified nearly 30 different HAAs in various processed foods, which can be broadly classified into two groups based on their chemical structures and the temperature at which they are formed: amino-imidazoazaarenes (AIAs) and amino-carbolines (ACs) (Alaejos and Afonso 2011). AIAs, characterized by an N-methyl-aminoimidazole structure, are primarily produced at typical cooking temperatures ranging from 100 to 300 °C, including quinolines, quinoxalines, and pyridines (Fig. 2A) (Dong et al. 2020). On the other hand, ACs are typically generated at temperatures exceeding 300 °C, which encompass α-carbolines, β-carbolines, γ-carbolines, δ-carbolines, and phenyl pyridines (Fig. 2B) (Dong et al. 2020). Additionally, recent discoveries have unveiled new HAAs in meat products, such as 2-amino-3,7-dimethylimidazo[4,5-f]quinoxaline (7-MeIQx) and 2-amino-1,7,9-trimethylimidazo[4,5-g]quinoxaline (7,9-DiMeIgQx), but their specific production conditions and properties are still not fully understood (Ni et al. 2008). Studies have indicated that HAAs significantly contribute to the occurrence and progression of human diseases (Bulanda and Janoszka 2022). The International Agency for Research on Cancer (IARC) has classified 2-amino-3,4-dimethylimidazo[4,5-f]quinoline (MeIQ), 2-Amino-3,8-dimethylimidazo[4,5-f]quinoxaline (MeIQx), 2-amino-1-methyl-6-phenylimidazo[4,5-b]pyridine (PhIP), 2-amino-9H-pyrido[2,3-b]indole (AαC), 2-amino-3-methyl-9H-pyrido[2,3-b]indole (MeAαC), 1,4-dimethyl-5H-pyrido[4,3-b]indol-3-amine (Trp-P-1), 3-amino-1-methyl-5H-pyrido[4,3-b]indole (Trp-P-2), 2-amino-6-methyldipyrido[1,2-a:3′,2′-d]imidazole (Glu-P-1), and 2-aminodipyrido[1,2-a:3′,2′-d]imidazole (Glu-P-2) as probable (2B) human carcinogens and 2-amino-3-methyl-3H-imidazo[4,5-f]quinoline (IQ) as a probable (2A) human carcinogen (World Health Organization 1993).

This review brings several novel aspects to the field compared to other studies (Table S1). First, it provides a comprehensive summary of the inhibition mechanism of HAA formation, covering the entire process from controlling precursors to regulating intermediates and promoting metabolism. This holistic approach sets it apart from studies that focus on specific aspects or mechanisms. Additionally, the review highlights the interconnected effects and interactions that lead to the formation of various hazardous substances during the thermal processing of food. By considering these linkage effects, it offers a unique perspective on the broader implications and potential consequences beyond HAAs formation alone. Furthermore, the review acknowledges the dual role of the Maillard reaction in generating both HAAs and unique flavors during thermal processing. It explores the effects of inhibiting HAAs formation on flavor substances, providing a comprehensive understanding of the impact of HAAs mitigation strategies on the sensory aspects of food. It also takes into account the rapid advancements in HAAs detection technology. It discusses not only the application status of conventional methods but also newer techniques for the pretreatment and detection of HAAs. This up-to-date perspective on the analytical aspects of HAAs research ensures the inclusion of the latest developments in the field. Moreover, the review extensively illustrates the distribution of HAAs in different thermally processed meats prepared through various cooking methods and examines their relative toxicity. In summary, this review provides a comprehensive synthesis of previous research on HAAs, serving as a theoretical foundation for effectively preventing the generation of HAAs during meat cooking processes and reducing associated health risks. Moreover, it establishes a theoretical framework for future investigations into targeted control technologies for HAAs formation in meat products and provides valuable insights into the variations in HAAs occurrence and their potential health implications.

## 2 Formation of HAAs

HAAs are mainly formed via the Maillard reaction and are influenced by various factors (Nadeem et al. 2021). Due to the complex chemical reactions involved, it is challenging to precisely explain the synthesis of HAAs in meat products. Although various HAAs have been isolated and identified in meat products and chemical model systems, only some formation mechanisms of HAAs have been confirmed.

### 2.1 Formation of AIAs

The generation mechanisms of AIAs, due to their diverse and complex structures, are not fully understood. Current research suggests that the formation of AIAs may occur through the combined action of reactive carbonyl species and free radicals (Zamora and Hidalgo 2020). The formation of AIAs typically involves the reaction of creatinine with ammonia and specific reactive carbonyl species, which act as limiting reagents for HAA formation and originate from various sources (Kizil, Oz, and Besler 2011).

**Fig. 3:**
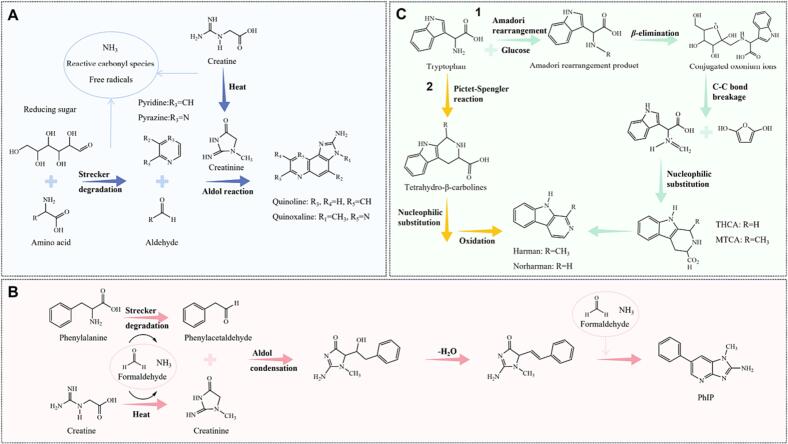
Proposed mechanisms of HAAs formation. (A) Proposed formation mechanism of quinoline/quinoxaline. (B) Proposed formation mechanism of PhIP. (C) Proposed formation mechanism of β-carbolines.

Creatine/creatinine, reducing sugars, and amino acids are the primary precursors for quinolines and quinoxalines (Skog 1993). The similar possible pathways for their generation can be summarized as follows (Fig. 3A): (a) Reducing sugars and amino acids undergo Strecker degradation, forming pyridine and pyrazine, while creatine is thermally degraded to creatinine; (b) Quinolines or quinoxalines are formed through the Aldol reaction of pyrazine or pyridine derivatives with compounds such as creatinine and aldehydes, which are generated in the previous stage (Kizil, Oz, and Besler 2011, Zamora and Hidalgo 2020). The difference between the formation of quinoline and quinoxaline lies in the reaction of pyridine or pyrazine with creatinine (Zamora and Hidalgo 2020). Additionally, alkylpyridine radicals and dialkylpyrazine radicals, formed by the oxidation of reducing sugars and amino acids at high temperatures, may directly react with aldehydes and creatinine to produce different types of quinoline or quinoxaline, respectively (Pearson et al. 1992). However, the lack of validated steps has led to controversy surrounding this mechanism.

Few studies have focused on the formation mechanisms of 2-amino-1,6-dimethylimidazo[4,5-b]pyrifine (DMIP) and 2-amino-1,5,6-trimethylimidazo[4,5-b]pyridine (1,5,6-TMIP) in pyridines (Felton et al. 1999). The formation mechanism of PhIP, on the other hand, has been well demonstrated through isotope labeling and is widely accepted, although it deviates from the typical HAA formation pathway (Zamora and Hidalgo 2015). The proposed formation mechanism of PhIP can be summarized in four steps (Fig. 3B): (a) Phenylalanine undergoes Strecker degradation to produce phenylacetaldehyde, while creatine thermally degrades to form creatinine. (b) Phenylacetaldehyde and creatinine undergo aldol condensation and subsequent dehydration. (c) Thermal reactions degrade phenylalanine, creatine, and carbohydrates to generate formaldehyde and ammonia. (d) Formaldehyde and ammonia combine with intermediates to form a complete pyridine ring (Zamora and Hidalgo 2015). Additionally, although reducing sugars such as glucose do not directly participate in PhIP formation, they still play a significant role in the process (Skog 1993).

Consequently, the formation of AIAs cannot be attributed solely to the Maillard reaction but rather to any process that generates the necessary reactive carbonyls and free radicals. Furthermore, the mechanisms proposed in this context are not sufficiently precise and may have certain missing steps. Therefore, further investigation is required to fully understand the comprehensive pathways of HAAs formation.

### 2.2 Formation of ACs

Typically, amino acid precursors, such as tryptophan, phenylalanine, glutamate, or ornithine, have the potential to generate active radicals and deamination or decarboxylation products at elevated temperatures, leading to the production of ACs (Murkovic, 2004, Alaejos and Afonso, 2011). Notably, casein and glycinin are also significant precursors in this process (Alaejos and Afonso 2011).

In comparison to other ACs, β-carbolines (Harman and Norharman) have the ability to form at lower temperatures and are widely found in various foods (Xue et al. 2020). Consequently, their formation mechanism has been extensively studied and reported. Tryptophan and glucose play crucial roles as precursors of β-carbolines (Gibis 2016). The synthesis of β-carbolines involves both reactive carbonyl species and free radicals. Acetaldehyde and α-keto acid, which are produced during the Maillard reaction, are key intermediates in the formation of β-carboline. Electron spin resonance (ESR) and hyperfine coupling constant analyses have revealed the involvement of alkyl radicals in the process (Xue et al. 2020). Based on this evidence, the formation mechanism of β-carbolines has been elucidated and widely accepted (Fig. 3C-2): (a) tryptophan generates the Amadori rearrangement product (ARP) in the presence of glucose; (b) a β-elimination reaction facilitated by epoxide lone-pair electrons leads to the formation of conjugated oxonium ions; and (c) the intermediates undergo intramolecular nucleophilic substitution after C—C bond breakage, resulting in the formation of β-carboline (Yaylayan, Jocelyn, and Laing 1990). Additionally, some researchers have proposed the involvement of tryptophan cyclization (Pictet-Spengler reaction) in the generation of tetrahydro-β-carbolines (THβC), which are further oxidized and undergo intramolecular nucleophilic substitution to yield β-carbolines (Fig. 3C-1) (Dong et al. 2020).

## 3 Distribution of HAAs in thermally processed meat

The relationship between diet and the formation of HAAs is deeply interconnected. In 1964, Lijinsky et al. first proposed the hypothesis that mutagenic compounds might be produced in thermally processed meat, and subsequent research confirmed that these mutagenic compounds in beef and fish were aromatic hydrocarbons (Lijinsky and Shubik 1964). It was later discovered that HAAs are primarily generated in protein-rich foods subjected to heat, such as meat, cooking residues, and cooking oil fumes. Furthermore, HAAs have been found in various sources, including coffee, alcoholic beverages, environmental sources such as cigarette smoke, rivers, and rain, as well as within the human body in substances such as hair, urine, and breast milk (Bulanda and Janoszka 2022). Given the widespread distribution of HAAs, their ingestion poses a significant challenge. Therefore, it is crucial to gain a comprehensive understanding of the distribution of HAAs in meat products to effectively mitigate the risk of their ingestion by humans.

The levels of HAAs in thermally processed meat vary significantly due to the diverse cooking and processing methods employed in different countries and regions. Additionally, other processing techniques and the composition of meat matrices can influence the types and quantities of HAAs present (Cheng et al., 2019, Yao et al., 2013). The analysis results presented in Table 1 demonstrate the presence of a wide range of HAAs in meat products, with notable variations observed among different matrices and processing methods. Carcinogenic HAAs, such as IQ, MeIQ, MeIQx, PhIP, AαC, MeAαC, Trp-P-1, and Trp-P-2, are commonly detected in various matrices, posing significant food safety risks. On the other hand, Glu-P-1 and Glu-P-2, also classified as Group 2B carcinogens, have received limited attention in existing research, possibly due to the scarcity of studies focused on these specific HAAs. Additionally, although Norharman and Harman are not classified as carcinogenic by the IARC, they are readily formed and abundantly present in all thermally processed meat, indicating the need for further investigation and attention to their potential implications.

**Table 1:** Types and contents of HAAs detected in thermally processed meats.

Category	Type	Processing Methods	Type of HAAs	Highest HAA content (ng/mL)	Total Content (ng/mL)	References
Livestock	Beef	Grill	Harman > PhIP > 4,8-DiMeIQx > MeIQx > Trp-P-2 > Norharman > MeIQ > Phe-P-1 > IQ > AαC	Harman (5.93–19.53)	4.83–89.28	(Szterk 2015)
			MeIQx > PhIP > AαC > Trp-P-1 > MeAαC	MeIQx (1.63 ± 0.57)		(Viegas et al. 2012)
			PhIP > 8-MeIQx > DMIP > IQx > Norharman > 1,5,6-TMIP > Harman > IQ[4,5-b] > 4,8-DiMeIQx > MeIQ > IQ > Phe-P-1	PhIP (18.14 ± 2.02)		(Yan et al. 2017)
		Fry	PhIP > Trp-P-1 > AαC > MeAαC > Trp-P-2 > Norharman > Harman > 4,8-DiMeIQx > Glu-P-2 > Glu-P-1 > MeIQx > MeIQ	PhIP (67.48 ± 6.99–116.90 ± 33.34)	1.22–850.76	(Dong, Lee, and Shin 2011)
			Harman > MeIQx > Norharman > PhIP	Harman (0.60 ± 0.01–1.10 ± 0.02)		(Gibis and Weiss 2012)
		Roast	MeIQx > Norharman > PhIP > Harman > 4,8-DiMeIQx > DMIP Harman > Norharman > IQ > MeAαC > MeIQ > AαC > DMIP > MeIQx > Phe-P-1 > 4,8-DiMeIQx > IQ[4,5-b]	MeIQx (4.08 ± 0.18)Harman (866.20 ± 11.64)	6.01–1679.51	(Chen et al. 2017)
		Boil	PhIP > 7,8-DiMeIQx > AαC > MeIQ > 4,8-DiMeIQx > IQx	PhIP (ND-2.94)	0.07–32.98	(Oz et al. 2016)
		Microwave	PhIP > MeIQx > DiMeIQx > IQ	PhIP (0.70–13.30)	2.30–20.60	(Felton et al. 1994)
		Marinate	Harman > Norharman > IQ > MeIQ > MeIQx	Harman (254.89 ± 0.81)	24.23–480.45	(Wang et al. 2017)
	Pork	Marinate	Harman > Norharman > IQ > MeIQ > MeIQx	Harman (189.11 ± 0.65)	0.06–345.23	(Wang et al. 2017)
			IQ > Harman > Norharman > AαC	IQ (ND-22.46 ± 1.80)		(Guo et al. 2020)
			PhIP > 4,8-DiMeIQx > MeIQx > IQ > AαC > Trp-P-1 > MeIQ	PhIP (0.44–2.64)		(Lan and Chen 2002)
			Harman > Norharman > PhIP > Trp-P-1	Harman (10.90 ± 0.54–29.23 ± 0.59)		(Chang et al. 2018)
			MeIQx > Trp-P-1 > PhIP > 4,8-DiMeIQx > AαC > IQ > MeIQ	MeIQx (1.44–8.85)		(Lan, Kao, and Chen 2004)
			PhIP > 8-MeIQx	PhIP (4.59 ± 0.72)		(Janoszka 2010)
		Fry	PhIP > MeIQx > 4,8-DiMeIQx	PhIP (5.32–18.40)	ND-43.71	(Zhang et al. 2013)
			Harman > Norharman	Harman (2.60 ± 0.03–12.47 ± 0.35)		(Pan et al. 2019)
			MeIQ > 8-MeIQx > 4,8-DiMeIQx	MeIQ (9.28 ± 0.82)		(Janoszka 2010)
			PhIP > Trp-P-1 > DiMeIQx > MeIQx	PhIP (0.04–32.00)		(Skog et al. 1997)
		Grill	Norharman > 8-MeIQx > Harman > AαC > IQx	Norharman (0.08 ± 0.01–0.80 ± 0.04)	0.16–1.61	(Chang et al. 2018)
			MeIQ > 8-MeIQx > PhIP	MeIQ (6.28 ± 1.55)		(Janoszka 2010)
			7,8-DiMeIQx > PhIP > 4,8-DiMeIQx > Norharman	7,8-DiMeIQx (ND-1.20)		(Bula et al. 2019)
		Roast	PhIP > 8-MeIQx > 1,5,6-TMIP > Norharman > Harman > DMIP > 4,8-DiMeIQx > MeIQ > Phe-P-1 > IQ[4,5-b] PhIP > DMIP > 8-MeIQx > 4,8-DiMeIQx > 1,5,6-TMIP > Norharman > Phe-P-1 > MeIQ > IQ[4,5-b] > Harman > IQ	PhIP (12.94 ± 1.25–114.30 ± 14.85)	2.81–306.00	(Yan et al. 2017)
			Norharman > Harman > Glu-P-1 > MeAαC > PhIP > AαC > 1,5,6-TMIP > MeIQ > IQ[4,5-b] > DMIP > Phe-P-1	Norharman (72.74 ± 0.44–84.59 ± 0.48)		(DiaodiaoYang et al. 2020)
			Harman > Norharman > AαC > DMIP > PhIP > Phe-P-1	Harman (147.00 ± 2.24–159.00 ± 2.52)		(Yang et al. 2019)
		Smoke	Norharman > Harman > AαC > PhIP > DMIP > Phe-P-1 > IQ[4,5-b]	Norharman (146.00 ± 0.50–154.00 ± 3.32)	30.26–422.00	(Yang et al. 2019)
			Norharman > Harman > MeAαC > MeIQ > IQ > IQx > 7,8-DiMeIQx > PhIP	Norharman (21.92 ± 1.69–313.39 ± 3.33)		(Yin et al. 2021)
		Floss	Norharman > Harman > AαC > PhIP > MeAαC > 4,8-DiMeIQx	Norharman (5.12 ± 0.21–14.98 ± 0.15)	34.21–950.96	(Pan et al. 2019)
			Norharman > Harman > Trp-P-2 > PhIP > AαC > Glu-P-2 > MeAαC	Norharman (35.40 ± 11.54–50.63 ± 10.47)		(Liao, Xu, and Zhou 2009)
			Glu-P-1 > Harman > IQ > Norharman Harman > IQ > Norharman > Trp-P-2	Glu-P-1 (342.19–718.46)Harman (149.73–254.81)		(Jian et al. 2019)
	Lamb	Marinate	Harman > Norharman > IQ > MeIQ > MeIQx	Harman (158.12 ± 0.46)	291.81	(Wang et al. 2017)
		Smoke	Norharman > Harman > AαC	Norharman (1.33–2.99)	2.74–5.42	(Hou et al. 2017)
		Roast	PhIP > Harman > Norharman > IQ > MeIQx > AαC > MeAαC > 4,8-DiMeIQx	PhIP (0.59 ± 0.22–97.13 ± 11.29)	4.39–123.15	(Guo et al. 2014)
			Phe-P-1 > IFP > MeIQ > Glu-P-2 > Harman > Norharman	Phe-P-1 (5.03 ± 1.46–8.47 ± 1.06)		(Xiao et al. 2020)
		Fry	PhIP > MeAαC > Harman > 4,8-DiMeIQx > Norharman > MeIQx PhIP > AαC > Norharman > MeIQx > Harman > Trp-P-2 > IQ	PhIP (0.18 ± 0.02–32.24 ± 5.68)	ND-43.24	(Guo et al. 2014)
			PhIP > DiMeIQx > MeIQx	PhIP (0.01–2.30)		(Skog et al. 1997)
		Stew	Harman > Norharman > 4,8-DiMeIQx > MeIQx > IQ	Harman (27.89 ± 3.12–58.59 ± 3.35)	51.07–120.32	(Guo et al. 2014)
		Grill	Harman > Norharman > DMIP > AαC > PhIP	Harman (0.73–65.07)	2.43–94.02	(Sun et al. 2010)
Poultry	Chicken	Barbecue	PhIP > Trp-P-1 > 4,8-DiMeIQx > MeIQx > MeAαC > AαC	PhIP (15.22 ± 2.83)	1.97–31.93	(Viegas et al. 2012)
			Norharman > Harman > MeIQx > 4,8-DiMeIQx > PhIP	Norharman (1.5 ± 0.1–12.9 ± 0.40)		(Solyakov and Skog 2002)
			Norharman > Harman > PhIP > AαC > Trp-P-2 > 4,8-DiMeIQx > MeAαC > Trp-P-1 > MeIQx	Norharman (32.18 ± 3.76)		(Liao et al. 2010)
		Fry	Norharman > PhIP > 4,8-DiMeIQx > AαC > MeAαC > Trp-P-1 > Trp-P-2 > MeIQ > MeIQx > Harman > 7,8-DiMeIQx > Glu-P-1 > Glu-P-2 > Tri-MeIQx	Norharman (3.33 ± 0.75–130.53 ± 12.11)	ND-826.53	(Dong, Lee, and Shin 2011)
			Harman > Norharman > PhIP > 4,8-DiMeIQx > 8-MeIQx	Harman (1.24 ± 0.05–29.92 ± 1.03)		(Chang et al. 2018)
			Norharman > Harman > Trp-P-1	Norharman (0.54 ± 0.18–1.41 ± 0.36)		(Yao et al. 2013)
			PhIP > Norharman > Harman > MeIQx > 4,8-DiMeIQx	PhIP (0.10–38.20 ± 2.00)		(Solyakov and Skog 2002)
			PhIP > MeIQx > Harman > IQ > 4,8-DiMeIQx > Norharman > AαC > MeAαC Harman > Norharman > PhIP > MeIQx > 4,8-DiMeIQx > AαC > MeAαC	PhIP (18.33 ± 3.63) Harman (12.32 ± 1.83)		(Liao et al. 2010)
			PhIP > Norharman > Harman > MeIQx > 4,8-DiMeIQx	PhIP (72.00 ± 9.00)		(Busquets et al. 2006)
			IQ > 7,8-DiMeIQx > MeIQ > MeIQx > 4,8-DiMeIQx > IQx	IQ (0.26–3.53)		(Oz, Kaban, and Kaya 2010)
			IQ > 4,8-DiMeIQx > Norharman > MeIQ > Harman > MeIQx > PhIP	IQ (0.86 ± 0.19–17.2 ± 1.35)		(Cheng et al. 2021)
			PhIP > DiMeIQx > MeIQx	PhIP (0.02–10.00)		(Skog et al. 1997)
		Smoke	Norharman > Harman > PhIP	Norharman (1.83 ± 0.80–10.64 ± 1.34)	3.306–372.56	(Chang et al. 2018)
			Norharman > Harman > MeAαC > PhIP > MeIQ > IQ > IQx > 7,8-DiMeIQx	Norharman (44.23 ± 3.10–293.19 ± 14.66)		(Zhang, Du, et al. 2020)
		Marinate	Harman > Norharman > IQ > MeIQ > MeIQx > PhIP	Harman (124.03 ± 0.52)	3.04–239.36	(Wang et al. 2017)
		Roast	Norharman > Harman > PhIP > 4,8-DiMeIQx > IQ[4,5-b]	Norharman (3.54 ± 0.58)	ND-201.24	(Yan et al. 2017)
			Norharman > Harman > MeAαC > PhIP > MeIQ > IQx > 7,8-DiMeIQx > IQ	Norharman (24.71 ± 1.48–166.15 ± 9.97)		(Zhang, Du, et al. 2020)
			Harman > PhIP > Norharman > MeIQx > 4,8-DiMIQx	Harman (0.10–3.30 ± 0.20)		(Solyakov and Skog 2002)
			Norharman > Harman > AαC > MeAαC > PhIP > Trp-P-1 > Trp-P-2	Norharman (3.05 ± 0.40)		(Liao et al. 2010)
			Harman > Norharman > isoIQ > 8-MeIQx > Trp-P-2 > PhIP > DMIP > IFP > 7,8-DiMeIQx	Harman (0.27 ± 0.20–23.03 ± 4.32)		(Hsu and Chen 2020)
			IQ > 4,8DiMeIQx > IQx	IQ (0.30–0.54)		(Oz, Kaban, and Kaya 2010)
		Braize	IQ > Norharman > MeIQx > Harman > 4,8-DiMeIQx > PhIP	IQ (ND-4.74 ± 0.73)	0.66–96.98	(Cheng et al. 2020)
			Norharman > Harman > Trp-P-1	Norharman (0.48 ± 0.19–1.33 ± 0.31)		(Yao et al. 2013)
			Norharman > Harman	Norharman (0.40 ± 0.20)		(Solyakov and Skog 2002)
			IQ > MeIQ > Harman > MeIQX > 4,8-DiMeIQx > Norharman > AαC > PhIP	IQ (2.61 ± 0.08–13.5 ± 1.38)		(Cheng et al. 2019)
		Microwave	Harman > Norharman > Trp-P-1 > Trp-P-2 > MeAαC > AαC	Harman (0.15 ± 0.04–2.84 ± 0.33)	0.10–8.21	(Chiu, Yang, and Chen 1998)
			IQ > 4,8-DiMeIQx	IQ (0.76–7.37)		(Oz, Kaban, and Kaya 2010)
		Grill	IQ > MeIQ > MeIQx > 7,8-DiMeIQx > IQx > 4,8-DiMeQx	IQ (0.26–1.11)	111.81	(Liao et al. 2010)
	Duck	Boil	Norharman > Harman	Norharman (0.23 ± 0.03–0.50 ± 0.11)	0.28–254.92	(Liao et al. 2012)
		Roast	Norharman > Harman > AαC > MeAαC > Trp-P-2 > Trp-P-1	Norharman (6.12 ± 1.19)	3.52–88.52	(Liao et al. 2010)
			Norharman > Harman > MeIQx > 4,8-DiMeIQx > Trp-P-2	Norharman (2.00 ± 0.31–18.07 ± 3.76)		(Liao et al. 2012)
		Fry	PhIP > Harman > Norharman > IQ > MeIQx > 4,8-DiMeIQx > AαC > MeAαC > Trp-P-2 > Trp-P-1 Harman > Norharman > 4,8-DiMeIQx > PhIP > MeIQx > AαC > MeAαC	PhIP (21.88 ± 3.13)Harman (6.03 ± 1.48)	0.66–97.71	(Liao et al. 2010)
			Norharman > Harman > PhIP > AαC > MeAαC > MeIQx > IQ > 4,8-DiMeIQx > Trp-P-2 > Trp-P-1 Norharman > Harman > PhIP > 4,8-DiMeIQx > IQ > MeIQx > Trp-P-1 > Trp-P-2	Norharman (0.38 ± 0.11–28.07 ± 3.36)		(Liao et al. 2012)
		Grill	PhIP > Harman > Norharman > IQ > MeIQx > 4,8-DiMeIQx > AαC > Trp-P-2 > MeAαC > Trp-P-1	PhIP (11.80 ± 1.66)	15.92–81.50	(Liao et al. 2010)
			Norharman > Harman > PhIP > AαC > 4,8-DiMeIQx > MeAαC > MeIQx > IQ > Trp-P-2 > Trp-P-1	Norharman (8.01 ± 1.83–33.03 ± 4.47)		(Liao et al. 2012)
		Microwave	Norharman > Harman > MeAαC > AαC > Trp-P-1 > Trp-P-2	Norharman (0.11 ± 0.03–0.53 ± 0.10)		(Liao et al. 2012)
		Boil	IQ	IQ (0.92–2.42)	0.92–2.42	(Oz, Kızıl, and Çelık 2016)
		Grill	IQ > MeIQ > 7,8-DiMeIQx > MeIQx > IQx > 4,8-DiMeIQx	IQ (0.20–0.39)	0.34–0.83	(Oz, Kızıl, and Çelık 2016)
		Fry	IQ > MeIQ > AαC > 7,8-DiMeIQx	IQ (0.18–1.12)	0.18–1.12	(Oz, Kızıl, and Çelık 2016)
		Microwave	IQ > MeAαC > AαC > 7,8-DiMeIQx > MeIQ > MeIQx > 4,8-DiMeIQx > IQx	IQ (1.08–1.44)	2.20–2.36	(Oz, Kızıl, and Çelık 2016)
Seafood	Pisces	Grill	PhIP > MeIQx > Trp-P-1 > MeAαC > AαC	PhIP (7.76 ± 2.04)	0.02–14.03	(Viegas et al. 2012)
			DMIP > PhIP > 8-MeIQx > IQx > 4,8-DiMeIQx > IQ[4,5-b] > AαC > 1,5,6-TMIP	DMIP (3.92 ± 0.39)		(Yan et al. 2017)
			Norharman > Harman	Norharman (1.80 ± 0.25–1.91 ± 0.14)		(Chang et al. 2018)
			IQ > 4,8-DiMeIQx > MeIQ > MeIQx > 7,8-DiMeIQx > IQx	IQ (0.58–2.56)		(Oz, Kaban, and Kaya 2010)
		Smoke	Norharman > Harman > Glu-P-1 > 8-MeIQx > DMIP > AαC > PhIP > IQ[4,5-b] > MeAαC > 4,8-DiMeIQx > 1,5,6-TMIP > MeIQ > IQ	Norharman (18.88 ± 2.62)	51.04	(Yan et al. 2017)
		Microwave	IQ > 4,8-DiMeIQx	IQ (3.99–17.84)	0.24–18.09	(Oz, Kaban, and Kaya 2010)
		Oven	IQ > IQx > 4,8-DiMeIQx	IQ (0.34–4.28)	0.28–4.28	(Oz, Kaban, and Kaya 2010)
		Fry	IQ > MeIQ > 7,8-DiMeIQx > IQx	IQ (0.27–5.97)	ND-211.80	(Oz, Kaban, and Kaya 2010)
			Salmon: PhIP > DMIP > Norharman > Harman > MeIQx > 4,8-DiMeIQx > IQ Tuna: Norharman > 4,8-DiMeIQx > MeIQx > PhIP > DMIP > Harman Sardine: Norharman > Harman > PhIP > MeIQx > DMIP > 4,8-DiMeIQx Swordfish: PhIP > Norharman > DMIP > Harman > MeIQx > 4,8-DiMeIQx Hake: PhIP > Norharman > DMIP > Harman > MeAαC > AαC Cod: PhIP > Norharman > DMIP > MeIQx > Harman > MeIQ > IQ Angler fish: Norharman > DMIP > PhIP > MeIQx > 4,8-DiMeIQx > MeAαC > IQ Sole: DMIP > Norharman > PhIP > Harman	PhIP (0.70 ± 0.01–121.00 ± 0.70)Norharman (2.10 ± 0.40–51.30 ± 3.90)DMIP (0.50 ± 0.05–37.80 ± 2.80)		(Khan et al. 2013)
			Cod fillet: PhIP > MeIQx > Trp-P-1 Baltic herring: PhIP > MeIQx > Trp-P-1	PhIP (0.01–2.20)		(Skog et al. 1997)
			7,8-DiMeIQx > MeIQx > Norharman > Harman > PhIP > AαC	7,8-DiMeIQx (2.30 ± 0.09–7.88 ± 1.02)		(Wang et al. 2015)
	Cuttlefish	Fry	Squid: Norharman > DMIP > Harman > PhIP Cuttlefish: Norharman > Harman > DMIP > PhIP	Norharman (17.60 ± 1.50–25.50 ± 1.50)	42.37	(Khan et al. 2013)

Research on HAAs encompasses various types of meat; however, the level of research attention and progress varies across different categories. It is worth noting that most of the existing research on HAAs has predominantly focused on poultry and livestock products, while studies on seafood are limited. Given that seafood is a significant source of HAAs, further investigation in this area is necessary. The types of HAAs generated in different thermally processed meats and cooking methods also vary due to differences in precursor composition among different meat matrices. As indicated in Table 1, Norharman, Harman, and PhIP were the predominant HAAs observed in livestock, whereas Norharman, Harman, PhIP, and IQ were the most prevalent HAAs found in poultry.

The composition and species of HAAs undergo changes with different processing methods. The total contents of HAAs produced by various processing methods, such as microwaving, grilling, frying, roasting, braizing, and smoking, are summarized in Table 1. The results showed that the content of HAAs in meat processed by stronger heat treatment methods such as frying, grilling, and roasting was higher, while the content of HAAs produced by boiling and microwaving was lower. Charcoal grilling and frying have higher HAAs due to the higher temperatures. HAAs were also detected in commercially available products, with the highest contents in sauces and braized foods (Wang et al. 2017). Norharman and Harman are the major HAAs produced by high-temperature treatments (Table 1). High temperatures and longer heating durations can increase the production of HAAs (Liao et al. 2010). Meat soups and skins also contain more HAAs than meat because water-soluble precursors can transfer to the surface of the thermally processed meat and, subsequently, to the soup during continuous stewing (Yao et al. 2013). Controlling heating temperatures, minimizing high-temperature processing times, and reducing marinade intake should be prioritized in daily cooking to reduce the risk of HAA exposure to human health. In sum, it is imperative to understand the distribution of HAAs in meat products to reduce their effects on physical health.

## 4 Toxicity of HAAs

### 4.1 Carcinogenesis

Numerous animal experiments have provided evidence that HAAs are carcinogenic and capable of inducing tumors in various organs of mice, including the liver and intestine (Im et al., 2010, Papier et al., 2021). In rodents, the ingestion of certain AIAs, such as PhIP and MeIQ, in sufficient quantities can lead to lesions in squamous cells, mammary glands, colons, and other organs, ultimately resulting in tumor formation (Im, Choi, and Kim 2010). However, it is important to note that the daily intake of HAAs in humans is significantly lower than the amounts used in animal experiments. Therefore, the findings from long-term animal studies cannot be directly applied to establish a specific threshold for HAA ingestion in humans. Nevertheless, a study has indicated that a higher total daily intake of HAAs was associated with increased risks of colon, rectal, bladder, and kidney cancer, with MeIQx specifically linked to lung cancer risk (Augustsson et al. 1999). The study found that for every additional 10 ng of MeIQx intake, the risk of developing lung cancer increased by 5% (Augustsson et al. 1999).

The available epidemiological data on the association between HAAs and cancer risk are inconsistent, partly due to low exposure to HAAs or inadequate study designs to assess well-done meat intake and its impact on cancer risk. However, higher intakes of red meat and processed meat have been consistently linked to an increased risk of various diseases and different types of cancer, including colonic polyps, hemorrhagic stroke, colorectal cancer, and liver cancer (Papier et al. 2021). Many studies have specifically shown a positive association between high intakes of MeIQx and PhIP and the incidence of different types of cancer in humans, particularly colon cancer (Gibis 2016). Epidemiological studies have indicated that individuals with significantly higher meat cooking degrees resulting from high-temperature cooking have a 2.8 times higher probability of developing colorectal cancer than those with regular intake, and the risk of rectal cancer can be up to six times higher (Lo et al. 2020). Daily consumption of 50 g of processed meat has been associated with increased risks of various cancers, such as pancreatic, prostate, breast, and colon cancers, with increases in risk ranging from 4% to 19% (Szterk 2015). Given that it is nearly impossible to completely avoid regular ingestion of HAAs, investigating the potential relationship between HAAs and certain human cancers remains a prominent research topic in cancer institutions.

### 4.2 Mutagenicity

HAAs have been extensively studied for their mutagenic properties, affecting DNA synthesis in both animal and cellular experiments (Im, Choi, and Kim 2010). In mutagenicity tests such as the Ames test using *Salmonella typhimurium* with the S9 metabolic activation system, HAAs have shown mutagenic activity tens to thousands of times higher than that of PAHs, nitrites, aflatoxin B1, and benzopyrene (Alaejos and Afonso 2011). The relative mutagenicity of HAAs can vary depending on the detection methods employed. MeIQ exhibits the highest relative potency in *Salmonella typhimurium* strain TA98, IQ shows the highest mutagenicity in human hepatocellular carcinoma (HepG2) cells, and PhIP or MeIQ demonstrate the highest mutagenicity in Chinese hamster ovary systems (Bulanda and Janoszka 2022). Consequently, assessing the relative mutagenicity of HAAs and predicting their effects on humans upon ingestion of certain amounts becomes challenging.

Moreover, Harman and Norharman themselves do not possess direct carcinogenic effects due to the absence of exocyclic amino groups. However, they can enhance the genotoxicity of other HAAs, thereby facilitating the generation of mutations or acting as mutagens (Papier et al., 2021, Totsuka et al., 1999). Since the activation of most HAAs relies on metabolic enzymes, they are considered promutagens. Typically, HAAs are absorbed in the small intestine and subsequently transported to the liver for activation. Metabolic activation initiates with N-oxidation by P450 cytochromes(Chen et al. 2020). The reactive molecule N-acetoxy amine can form new polymers with DNA, leading to DNA damage, genetic mutations, cell proliferation, and eventually tumor formation. HAAs are also considered biomarkers for assessing cancer risk due to the formation of HAA-DNA adducts, which can increase the likelihood of developing cancer (Bulanda and Janoszka 2022). Epidemiological investigations have highlighted the significant association between HAAs and the incidence of pancreatic, prostate, lung, breast, colon, and gastric cancers (Guo et al. 2018). Therefore, conducting research on HAAs holds great significance for promoting overall physiological health.

### 4.3 Risk assessment

The comprehensive analysis in Table 1 reveals the widespread presence of HAAs in various meat products. Prolonged consumption of thermally processed meat containing HAAs can result in severe organ disorders and numerous detrimental effects (Zhang, Zhao, et al. 2020). Notably, red meat falls under Group 2A, categorized as probably carcinogenic to humans, while processed meat is classified as Class 1, recognized as carcinogenic to humans(Dong et al. 2020). Although the intake of HAAs varies across countries, an average individual consumes approximately 420 ng of HAAs per day, and the European Commission recommends a daily intake of less than 1 μg per person (Barzegar, Kamankesh, and Mohammadi 2019). While the association between HAA intake and human cancer remains a topic of controversy, given their potential mutagenicity, carcinogenicity, and widespread presence in food, HAAs have become a focal point of research in various fields.

## 5 Detection methods of HAAs

HAAs are extensively present in a diverse range of thermally processed meats and have adverse implications for physical health. As a result, it is essential to extract, analyze, and monitor HAAs from various meat matrices to ensure food safety.

### 5.1 Pretreatment

**Fig. 4:**
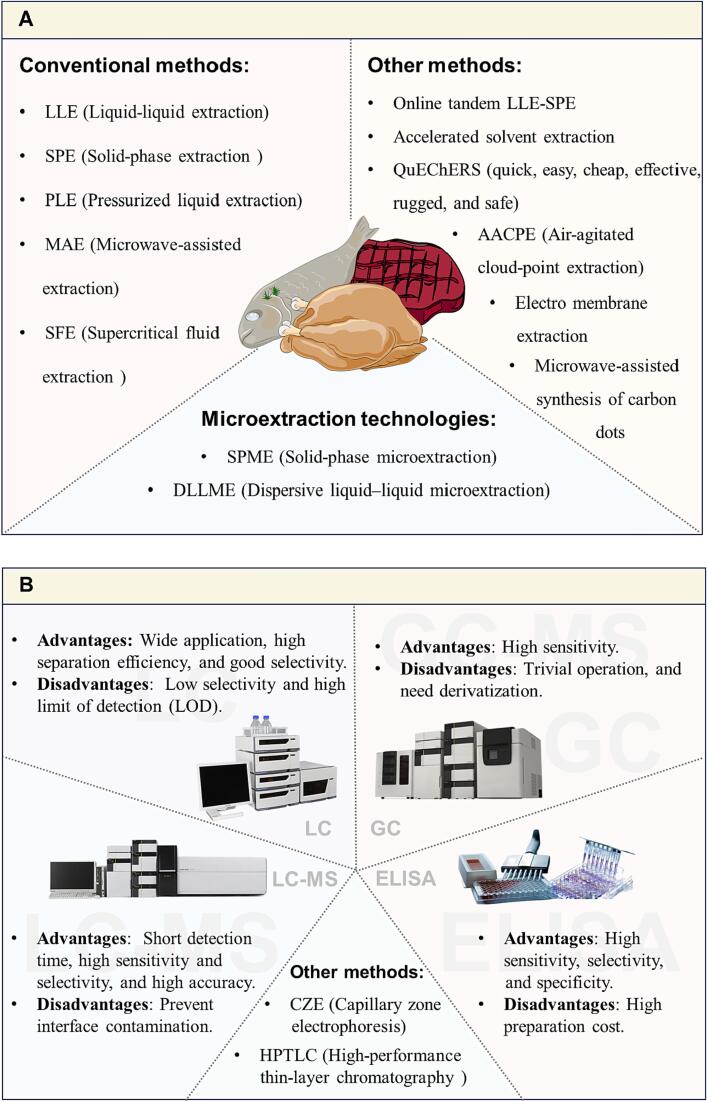
Various detection methods of HAAs. A: various pretreatment methods of HAAs; B: various detection methods of HAAs.

To ensure accurate measurement of HAAs in meat products, it is crucial to employ simple and effective sample preparation procedures. The pretreatment of HAAs involves two main aspects: extraction and purification (Chen et al. 2020). Currently, standard methods for extracting and purifying HAAs include various conventional techniques, such as liquid–liquid extraction (LLE), solid-phase extraction (SPE), pressurized liquid extraction (PLE), microwave-assisted extraction (MAE), and supercritical fluid extraction (SFE), as well as microextraction technologies, such as solid-phase microextraction (SPME) and dispersive liquid–liquid microextraction (DLLME) (Fig. 4A) (Barzegar, Kamankesh, and Mohammadi 2019). These methods contribute to the accurate and reliable analysis of HAAs in meat samples.

Traditional methods such as LLE and SPE have been widely used for extracting HAAs from various food matrices. LLE involves the use of two phases, organic and aqueous, in equal proportions for large-volume extraction, where the analytes are transferred from the aqueous phase to the organic phase. However, the drawbacks of LLE, including the need for large amounts of toxic solvents, lengthy processes, analyte dilution, and low recovery, limit its application (Aeenehvand et al. 2016). On the other hand, SPE is considered superior to LLE and offers optimized sensitivity and selectivity. It utilizes various sorbents, including silica gel and preparative columns, to enhance the extraction process (Barzegar et al. 2019). In addition to commonly used adsorbents such as diatomite, rayon, blue cotton, propyl sulfonic acid columns, strong cation exchange (SCX) columns, C18 reversed-phase columns, and Lichrolut EN, newer adsorbents such as magnetic carbon nanotubes and graphene composites have been identified to further improve HAAs extraction (Liang et al., 2020, Cai et al., 2017).

The extraction process of HAAs from meat products involves several steps. Initially, LLE is employed for extraction and homogenization using organic solvents such as acetone, ethyl acetate, and methanol or inorganic solvents such as water, hydrochloric acid, and sodium hydroxide. The corresponding extraction solvents are inorganic (hydrochloric acid) and organic (dichloromethane and ethyl acetate) (Gross and Grüter 1992). Subsequently, the homogenized liquid is combined with the absorbent material. SPE is then used for separation and purification, often utilizing cation exchange (MCX) and reversed-phase (C18) columns. The key steps in SPE include column activation, equilibration, sample loading, washing, drying, elution, spin drying of the eluent, reconstitution, and detection. These steps collectively enable effective extraction and purification of HAAs from meat samples.

PLE is a technique that relies on the diffusion of an extraction solvent at elevated temperatures (50–200 °C) and high pressures (1.0 × 10^4^-1.4 × 10^4^ kPa) to extract the target analyte from a solid sample. PLE offers several advantages over other methods, including time efficiency, reduced solvent consumption, automation, and improved extraction efficiency. Therefore, PLE is a highly suitable method for extracting HAAs from meat products.

SFE is a technique that utilizes supercritical carbon dioxide, which offers advantages such as safety, environmental friendliness, and ease of use, to extract target analytes. In an SFE system, when the fluid reaches a supercritical state, the target analyte can be dissolved and extracted by adjusting the temperature and pressure conditions. However, its ability to dissolve different substances and its overall suitability for extraction purposes still require further confirmation (Thiebaud et al. 1994).

MAE is a technique that involves heating the sample in a microwave field to facilitate the extraction and separation of the target analyte. By converting the microwave energy within the sample into heat energy, MAE enables the differentiation and extraction of HAAs. This method is known for its efficiency and ability to achieve high recovery rates (Mohammadi et al. 2016). Consequently, it has been widely adopted for extracting target analytes from complex solid matrices, particularly in the case of thermally processed meats (Ramezani et al. 2015).

Microextraction technologies have emerged as alternatives to overcome the limitations of traditional LLE and SPE methods. One such technology is SPME, which does not rely on an extraction solvent and differs from SPE in that it does not extract the entire analyte. The principle of SPME involves establishing an equilibrium distribution between the aqueous phase and an organic polymer phase coated on fused silica fibers, where adsorption and desorption take place (Zhang et al. 2015). The selection of an appropriate fiber coating is crucial for target analyte extraction. This method has gained significant attention due to its compatibility and feasibility. Another microextraction method is DLLME, which involves rapidly adding a mixture of dispersion solvent (water-organic miscible solvent) and extraction solvents (organic solvents) to an aqueous solution containing the analytes of interest. This process promotes the formation of a turbid solution, enabling rapid enrichment of the intended analyte due to the increased contact area between the two phases (Zamora and Hidalgo 2015). DLLME offers several advantages, including low solvent volume requirements, environmental friendliness, high recovery, high sensitivity, strong selectivity, cost-effectiveness, and short extraction time. Consequently, this technique has been utilized for the determination of HAAs in various food samples.

In addition to the previously mentioned separation methods, several other techniques have been utilized for the extraction of HAAs. These include online tandem LLE-SPE, accelerated solvent extraction, microsolid-phase extraction (micro-SPE), QuEChERS (quick, easy, cheap, effective, rugged, and safe), and electromembrane extraction (Ouyang et al., 2015, Zhang et al., 2015, Hsiao et al., 2017, Kamankesh et al., 2020). Air-agitated cloud-point extraction (AACPE) has also been employed for the extraction and preconcentration of four HAAs (MeIQ, 4,8-DiMeIQx, PhIP, and Harman) (Vichapong, Burakham, and Srijaranai 2016). This method has demonstrated successful application in the analysis of highly concentrated samples. Another innovative approach is the microwave-assisted synthesis of carbon dots, which is a novel nanotechnology used as an alternative to optimize the extraction process before chromatographic analysis. This technique involves the low-cost synthesis of fluorescent nanoparticles. By assessing the fluorescence quenching of carbon dots and measuring their lifespan in the presence of HAAs, this method provides an advanced means to quantify HAA formation (López et al. 2015).

### 5.2 Detection

For the accurate measurement of trace amounts of HAAs in complex thermally processed meat samples, the selection of a rapid, effective, selective, and sensitive detection method is crucial. Currently, several detection techniques (Fig. 4B) are commonly employed for the analysis of HAAs, including liquid chromatography (LC), liquid chromatography-mass spectrometry (LC-MS), gas chromatography (GC), and enzyme-linked immunosorbent assays (ELISA).

Since the late 20th century, chromatographic techniques have been utilized for quantifying HAAs in food. Among these techniques, high-performance liquid chromatography (HPLC) coupled with a detector based on the study by Gross and Grüter (1992) is the most widely employed method for analyzing and detecting HAAs (Gross and Grüter 1992). This approach significantly improves the selectivity and sensitivity of detection. The detector is connected to a computer data workstation, which is an HPLC system component that can record the electrical signals required to generate chromatograms on the display and to identify and quantify the concentration of sample components. Several different types of detectors have been developed to deal with different sample compounds with extremely different characteristics. Most HAAs exhibit UV or fluorescence absorption within the wavelength range of 260–275 nm, enabling their detection using HPLC with an ultraviolet–visible (UV–Vis) or fluorescence detector. If a compound does not possess any of these properties, evaporative light-scattering detector (ELSD), a more general detector type, is used. However, HPLC does have limitations. It has relatively low selectivity, making it challenging to completely separate the target analyte from complex matrices, resulting in potentially inaccurate results. Additionally, this method has a high limit of detection (LOD), making it difficult to detect trace amounts of HAAs present in food matrices. Therefore, the development of a more sensitive detection method is necessary.

In recent years, LC-MS has been increasingly utilized for the qualitative and quantitative analysis of HAAs. This method employs electrospray ionization (ESI), atmospheric pressure chemical ionization (APCI), or atmospheric pressure photoionization (APPI) to ionize target fragments, which are further identified by MS. HAAs contain amino groups or nitrogen atoms with varying pKa values, and their ionizable group quantity and position are diverse, leading to distinct chromatographic characteristics for different HAAs. LC separates the target analytes based on their molecular weight, while MS determines their structures. The combination of LC-MS offers higher sensitivity and selectivity, with LODs as low as pg. HPLC-MS is a rapidly evolving analysis method that effectively combines the efficient separation capability of LC for thermally unstable and high-boiling point substances with the analytical ability of MS for complex components, making it effective for analyzing complex organic mixtures (Xu et al. 2021). Moreover, MS application helps to prevent false positive and false negative results. Using available or presumed data on ion reactions, multiple reaction monitoring (MRM) is a mass spectrometry technique capable of specifically acquiring quantitative information in mass spectrometry. By integrating MRM with the highly sensitive single mass-to-charge ratio scan of a triple quadrupole mass spectrometry system, it becomes possible to achieve quantitative analysis of HAAs. Ultra-performance liquid chromatography-tandem mass spectrometry (UPLC-MS), an improved method based on HPLC, employs a fine particle size filler (1–2 μm) and a narrow inner diameter chromatographic column, resulting in high column efficiency and accelerated synchronous detection of HAAs, significantly enhancing detection efficiency (Oz and Kızıl 2012). This method substantially reduces the detection time and provides technical support for rapid analysis, particularly when analyzing numerous samples.

HAAs possess strong polarity and low volatility, which can lead to excessive retention on the chromatographic column, resulting in broad peaks or peak tailing in the chromatogram (Barzegar, Kamankesh, and Mohammadi 2019). To address this issue, it is necessary to reduce polarity and increase volatility and resolution through derivatization. GC is commonly employed to analyze low-polarity or nonpolar HAAs (Dong et al. 2020). According to the response to the detected substance, gas chromatography detectors can be divided into general detectors, including thermal conductivity detector (TCD), hydrogen flame ionization detector (FID), photoionization detector (PID), and selective detectors, including flame photometric detector (FPD), electron capture detector (ECD), nitrogen and phosphorus detector (NPD). However, only a few HAAs can be successfully derivatized, and accurate quantification becomes challenging when the HAA concentration in the sample is too low. As a result, GC is less commonly used for HAA detection. GC–MS combines the efficient separation capability of GC for complex substances with the qualitative, quantitative, and efficient analysis and identification abilities of MS. However, the complex derivatization process, poor reproducibility, and high injection temperature may lead to false results, thereby limiting its application in HAA detection (Mohammadi et al. 2016).

ELISA is another detection method that offers high sensitivity and selectivity for HAAs. Specific monoclonal antibodies targeting HAAs have been generated, and corresponding ELISA analysis techniques have been established, making this method a promising and appealing approach (Sheng et al. 2020). Enzyme-labeled antibodies can specifically bind to antigens or antibodies adsorbed on solid-phase carriers. After adding the substrate solution, the substrate can change the hydrogen donor contained in it from a colorless reducing form to a colored oxidizing form under the action of the enzyme, and then the color reaction occurs. The color reaction of the substrate is used to determine whether there is a corresponding immune response, and the gradation of color is proportional to the amount of the corresponding antibody or antigen in the sample, which can be quantitatively determined by the ELISA detector, so that the sensitivity of the enzymatic reaction and the specificity of the antigen–antibody reaction are combined. Despite its simplicity and relatively low cost, the development of monoclonal antibodies is a complex process. As a result, only a few monoclonal antibodies against HAAs, such as IQ, MeIQ, MeIQx, 4,8-DiMeIQx, and PhIP, have been successfully produced (Vanderlaan et al. 1988). The challenging commercialization process further limits the widespread adoption and advancement of this approach.

In addition to the aforementioned methods, high-performance thin-layer chromatography (HPTLC) and capillary zone electrophoresis (CZE) can also be employed for HAA detection. HPTLC offers several advantages, including low cost, minimal interference, and simplicity of operation. However, it has lower accuracy compared to HPLC and drawbacks such as time consumption and possible contamination of the target analytes. As a result, HPTLC is currently less commonly utilized for HAA detection (Jautz, Gibis, and Morlock 2008). On the other hand, CZE-MS technology combines the high selectivity and sensitivity of MS with the efficient separation and detection capabilities of CZE. It exhibits unique advantages in separating and detecting trace HAAs in complex food matrices and holds promising prospects for further development. Additionally, a fluorescence immunosensor has been applied for the simultaneous detection of eight HAAs, demonstrating its potential in HAA analysis (Zhang et al. 2021).

In summary, each detection method possesses its own strengths, limitations, and suitability (refer to Fig. 4B). Therefore, it is crucial to select an appropriate method based on the specific experimental subject, conditions, and detection limits, which will provide the necessary scientific and theoretical foundation for future research on HAAs.

## 6 Inhibition mechanism of HAAs

**Fig. 5:**
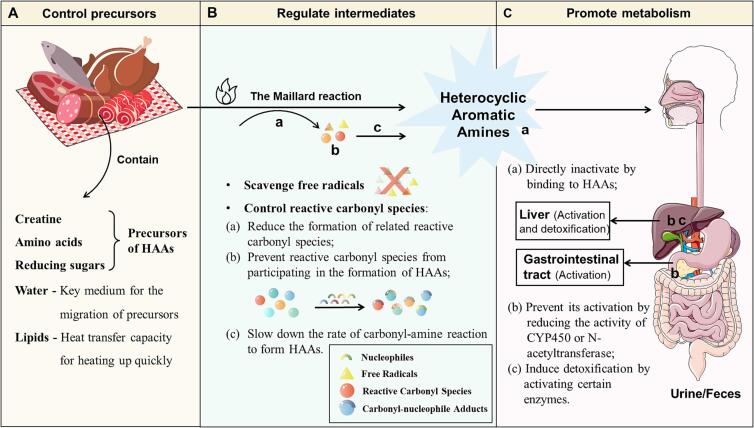
Inhibition mechanism of HAAs. A: by regulating precursors; B: by controlling intermediates (free radicals and reactive carbonyl species); C: by promoting the metabolism of HAAs.

Given the toxic effects of HAAs on human health, researchers have been exploring various methods to inhibit their production, such as modifying cooking techniques and introducing exogenous antioxidants (Chen et al. 2020). Currently, the inhibition mechanisms of HAAs in both chemical model systems and meat products mainly revolve around three key areas (Fig. 5): controlling precursors, regulating intermediates (including scavenging free radicals and controlling reactive carbonyl species), and promoting metabolism. These areas are crucial for providing scientific insights and support for future investigations into HAAs.

### 6.1 Controlling precursors

The formation of HAAs is greatly influenced by precursors, including sugars, amino acids, and creatine (creatinine), which play a crucial role in the production of HAAs. By controlling the type and content of these precursors, it is possible to reduce the formation of HAAs.

The current research places significant emphasis on the competitive inhibition of precursor reactions as a means to control the formation of HAAs. Creatinine, which is formed from the cyclization of creatine under high temperatures, serves as a crucial precursor for AIAs and is involved in the hydroxyl aldehyde reaction (Kizil et al., 2011). Additionally, the formation of imidazole rings, necessary for the formation of quinoxaline and quinoline, relies on creatinine. Therefore, inhibiting creatinine can effectively hinder the formation of HAAs (Zamora et al., 2020b, Hidalgo et al., 2021). When the carbohydrate content exceeds that of creatine, excess reducing sugars preferentially form 5-hydroxymethyl-2-furfural through the Maillard reaction, which inhibits HAA formation by reacting with creatinine (Dong et al. 2020). Furthermore, the addition reaction between compounds containing electrophilic groups and the nucleophile produced through creatinine enolization can impede HAA formation (LoPachin, Geohagen, and Nordstroem 2019). For instance, substances such as cinnamaldehyde in cinnamon, thymoquinone in black cumin, piperine in black pepper, curcumin, and allicene in artichoke, which contain electrophilic groups, readily form covalent adducts with electron-rich nucleophiles, thereby inhibiting HAA formation (Sepahpour et al., 2018, Tengilimoglu-Metin and Kizil, 2017, Oz, 2019).

Furthermore, controlling moisture and fat during the meat processing stage is another crucial approach to inhibit HAA formation by reducing the migration and heat transfer capacity of precursor substances. Moisture, as one of the primary constituents of meat products, serves as a critical medium for transferring HAA precursors (Alaejos and Afonso 2011). Excessive moisture content in meat products can lead to the transportation of internal precursors to the surface through excess water, thereby increasing HAA formation. Heating meat with higher fat content results in the production of more HAAs due to the enhanced heat transfer capability, where meat with higher fat content is more likely to reach the necessary thermal temperature for HAA production, and consequently, various precursors are more prone to react and form HAAs (Alaejos and Afonso, 2011, Pearson et al., 1992).

Effectively managing the formation of HAAs in meat products necessitates a comprehensive understanding of the intricate reaction system involved in their production. It is essential to control the precursors involved in HAA formation and gain clarity on the mechanisms by which these substances act.

### 6.2 Regulating intermediates

#### 6.2.1 Scavenging free radicals

The formation pathways of free radicals associated with the generation of HAAs can be described as follows: first, certain free radicals are produced through consecutive reduction reactions during the formation of N,N1-dialkylpyrazinium ions from glyoxal monoalkylimine; second, other free radicals are generated by forming a “biomolecular ring” and subsequently converting it into an enol of glycol-aldehyde alkylamine through oxidation processes.

Food-derived antioxidants, which are naturally abundant in phenolic compounds, have the ability to inhibit the formation of HAAs by scavenging free radicals in the reaction system (Hidalgo, Navarro, and Zamora 2018). Examples of such antioxidants include dried apple peels, green tea, olive oil, beer/red wine, rosemary, garlic, onion, red chili, paprika, ginger, turmeric, and black pepper, which have been shown to inhibit the formation of HAAs by scavenging pyrazine cation radicals or carbon-centered radicals in both chemical models and cooked meat (Wang et al., 2021, Zeng et al., 2018, Bao et al., 2020). Previous studies have suggested that the stronger the free radical scavenging activity is, the greater the inhibition of HAAs. However, it has been observed that naringin, despite showing weak free radical scavenging activity, exhibits strong inhibition of PhIP formation (Zamora, Alcon, and Hidalgo 2013). This indicates that free radical-related reactions may not be the rate-limiting step for PhIP formation in the model system. It is possible that other factors play a role in the inhibition of PhIP by naringin. Additionally, the correlation between quinic acid, sinapyl alcohol, naringenin, and the inhibition of HAAs was found to be weak (Wang et al. 2018). This suggests that the primary mechanism by which phenolic compounds inhibit HAAs is not solely based on free radical scavenging. Therefore, the free radical scavenging theory has certain limitations.

#### 6.2.2 Controlling reactive carbonyl species

Reactive carbonyl species, characterized by their reactive carbonyl structures, are crucial intermediate products in the Maillard reaction and play a significant role in the formation of HAAs. Controlling reactive carbonyl species can inhibit the formation of HAAs, and this inhibition mechanism can be understood from three perspectives: (a) reducing the formation of related reactive carbonyl species, (b) preventing the involvement of reactive carbonyl species in HAA formation, and (c) slowing the rate of the carbonyl–amine reaction during HAA formation (Hidalgo and Zamora 2022).

Reactive carbonyl species, which contribute to the formation of HAAs, can arise from various sources, such as carbohydrates, lipids, and amino acids. Consequently, inhibiting the production of these reactive carbonyl species is a potential strategy to hinder HAA formation. One common pathway for generating reactive carbonyl species is the Maillard reaction, which is particularly associated with the flavor and browning of food products. Another significant source of reactive carbonyl species is lipid oxidation. Under hypoxic conditions, lipids are less prone to oxidation, thereby restricting the formation of HAAs. By employing strategies to limit both the Maillard reaction and lipid oxidation, it becomes feasible to decrease the production of reactive carbonyl species and inhibit the formation of HAAs (Rannou et al. 2016).

Reactive carbonyl species, which are involved in the formation of HAAs, can readily react with nucleophiles such as amino acids, thiols, and antioxidants, leading to their consumption. As a result, any nucleophile capable of reacting with reactive carbonyl species associated with HAA formation or competitively inhibiting the carbonyl-creatinine reaction can influence HAA formation. Amines and thiols have been identified as inhibitors of HAAs, with examples including thiamine in garlic and onions, which can inhibit HAA formation by reacting with reactive carbonyl species (Hidalgo and Zamora 2022). Certain amino acids have shown similar efficacy, competitively reducing HAA production in roasted beef patties (Linghu et al. 2020). Some polyphenolic compounds are capable of eliminating intermediates and inhibiting HAA formation. Phenylacetaldehyde, an important intermediate in PhIP formation, can be captured by polyphenolic compounds such as epigallocatechin gallate (EGCG), quercetin, naringenin, and nor-carotene, resulting in the inhibition of PhIP formation (Zheng et al., 2016, Yu et al., 2016). Similarly, curcumin has been found to inhibit the formation of Harman and Norharman in braized pork by controlling carbonyl compounds involved in the formation of 1,2,3,4-tetrahydro-β-carboline-3-carboxylic acid (Wang et al. 2021).

Upon the production of reactive carbonyl species, they actively engage in the carbonyl–amine reaction, leading to the generation of HAAs. The preferential formation of specific compounds depends on factors such as reactant availability, reaction conditions, and activation energies of different reactions. Research indicates that the interaction between carbonyl and creatinine can be effectively decelerated by manipulating parameters such as pH, temperature, and heating duration during the carbonyl-ammonia reaction (Zamora et al., 2020b, Zamora et al., 2020a, Hidalgo et al., 2021). Considering the significant role of the carbonyl–amine reaction in HAA formation, conditions that favor the generation of reactive carbonyl species can promote HAA formation, and vice versa.

### 6.3 Promoting metabolism

Currently, there are various methods available to reduce the intake of HAAs. However, considering their widespread presence, it is inevitable that some HAAs may still enter the body. To mitigate the metabolic toxicity of HAAs within the body, the following approaches can be explored: (a) direct inactivation by binding to HAAs, (b) prevention of their activation by reducing the activity of CYP450 or N-acetyltransferase, and (c) induction of detoxification by activating specific enzymes (Meurillon and Engel 2016).

Certain compounds have demonstrated the ability to directly bind and deactivate HAAs. Chlorophyll, for instance, can limit the metabolism of HAAs by forming covalent bonds with them (Damašius et al. 2011). HAAs also exhibit binding affinity to the cell membrane of bacteria, leading to their inactivation or detoxification, consequently reducing DNA damage caused by HAAs. Isothiocyanates (ITCs) react with the amino groups of HAAs, resulting in the formation of thiourea. This reaction helps prevent the formation of genotoxic N2-hydroxyl derivatives and reduces the risk of cancer (Zhang et al. 2017). Modifying the digestibility of HAAs is another approach to reduce their bioaccessibility. For instance, when HAAs are absorbed by dietary fibers, they cannot be readily absorbed by the small intestine, thus mitigating their bioactivation in the liver and lowering the risk of cancer (Kim and Je 2014).

The activation process plays a pivotal role in the metabolism of HAAs within the body. Pathogenic bacteria produce enzymes that catalyze the formation of acrolein, which can react with HAAs in the gut. However, lactic acid bacteria have the ability to reduce the activity of these enzymes, thereby diminishing the carcinogenic risk (Mirsadeghi et al. 2019). Vegetables containing glucosinolates undergo digestion to produce metabolites that interact with activation enzymes, inhibiting cytochrome activities and preventing HAA activation, thus reducing DNA damage (Kim and Je 2014). Consumption of well-done cooked meat induces the production of CYP1A2, an enzyme responsible for HAA bioactivation. Dietary fibers and nonstarch polysaccharides also exhibit inhibitory effects on CYP450, a phase I enzyme involved in HAA metabolism (Meurillon and Engel 2016). Furthermore, certain fatty acids have the capacity to modulate CYP1A and inhibit prostaglandin H synthase, an enzyme involved in the bioactivation of HAAs, thus reducing their toxicity (Mirsadeghi et al. 2019).

Enzymes play a crucial role in the detoxification of HAAs, leading to their elimination through urine or feces. Coffee contains specific diterpenoids that activate detoxification enzymes, such as glutathione transferase, which helps reduce the formation of HAA-DNA adducts, particularly PhIP-DNA adducts. The induction of glucuronidase can enhance HAA genotoxicity by regenerating the parent HAA or the genotoxic N-hydroxylated HAA metabolite. Consequently, HAAs undergo conjugation with glucuronosyltransferases, allowing for their detoxification (Chen et al. 2020). Furthermore, certain natural compounds rich in flavonoids can activate the detoxification of HAAs by inducing the activity of detoxification enzymes. Xanthohumol, for instance, can modulate the expression of CYP450 and UGT1A1 enzymes to prevent DNA strand breakages induced by HAAs in HepG2 cells, which appears to be the primary mechanism underlying the protective effects of xanthohumol against HAA-induced genotoxicity (Meurillon and Engel 2016).

Research indicates that only a small portion, ranging from 0.5% to 6.0%, of HAAs in the human body remains unmetabolized (Meurillon and Engel 2016). Dietary factors have a significant impact on HAA metabolism, suggesting that regulating dietary components can be an effective approach to mitigate HAA metabolic toxicity (Alaejos and Afonso 2011). For instance, the consumption of cabbage alongside meat has been shown to limit the formation of HAAs on the outer layer of burgers and decrease the overall mutagenic activity of HAAs in cooked meat (Lewandowska et al. 2014). Additionally, the appropriate use of fats and fatty acids is crucial for reducing the carcinogenic effects of HAAs, emphasizing the importance of regulating cooking oil usage (Zamora, Alcon, and Hidalgo 2013). Certain dietary fibers, such as wheat bran and potato fiber, containing lignin can directly bind to HAAs, thereby reducing their absorption in the intestine (Persson et al. 2004). In conclusion, exerting control over HAA metabolism through dietary interventions is essential to mitigate the associated risks.

## 7 Effect of inhibiting the formation of HAAs on flavor substances

Throughout the heating process, various changes occur in thermally processed meat, including alterations in protein structure and other components. These changes contribute to the transformation of texture, appearance, flavor, and chemical properties, ultimately enhancing its palatability. However, it is important to note that alongside these desirable changes, the formation of flavor and processing hazards, such as HAAs, also takes place simultaneously.

### 7.1 Formation of flavor substances in thermally processed meat

The formation of flavor compounds in thermally processed meat involves the interplay of lipid oxidation, the Maillard reaction, and the degradation of vitamins and their byproducts. Volatile compounds derived from lipids are recognized as the key contributors to the distinct flavor characteristics. This is attributed to the significant variation in unsaturated fatty acids present in the fat deposits of different animals, leading to differences in the profiles of volatile carbonyls, which are major products of lipid degradation (Calkins and Hodgen 2007). Even though only a small fraction of fatty acids undergo oxidation among these species, their presence is sufficient to considerably influence the overall flavor of the meat (Khan, Jo, and Tariq 2015).

In thermally processed meat, the Maillard reaction is primarily responsible for the generation of aromatic compounds, which contribute to the overall flavor profile (Bailey et al. 1994). The flavor compounds formed through the Maillard reaction can be classified into three categories (Mottram 1994). The first category comprises flavor substances produced by the dehydration and fragmentation of sugars, including compounds such as furan, pyranones, and dicarbonyls. These compounds not only serve as important precursors for other flavor substances in the Maillard reaction but also have flavor properties themselves (Chen, Cui, and Feng 2022). The second category encompasses degradation products of amino acids, such as aldehydes, sulfides (e.g., hydrogen sulfide, methyl mercaptan), and nitrogen-containing compounds. These compounds are formed through the Strecker degradation reaction between amino acids and dicarbonyls (Teng et al. 2023). Furthermore, the Maillard reaction products mentioned above can undergo additional reactions. For instance, they can interact with amines, amino acids, hydrogen sulfide, thiols, acetaldehyde, and other aldehydes. These interactions lead to the formation of the third category of flavor substances, including pyrrole, imidazole, oxazole, thiazole, thiophene, and furan thiol (Chen, Cui, and Feng 2022).

### 7.2 The effect of different inhibition strategies on the formation of HAAs and flavor substances

The formation of HAAs is influenced by three main factors: precursor content, processing or cooking methods, and the presence of additional exogenous substances. To inhibit the formation of HAAs, three strategies can be employed: (a) choosing appropriate food types; (b) optimizing processing methods; and (c) incorporating exogenous inhibitors such as natural product extracts, antioxidants, or other substances. It is important to note that inhibiting the formation of HAAs through these methods may have an impact on the overall flavor of meat products.

#### 7.2.1 Rational selection of food types

The composition and content of HAAs in thermally processed meat products are significantly influenced by the meat matrices used. Table 1 demonstrates that beef and mutton typically have higher levels of HAAs than poultry and fish, indicating variations in HAA formation among different animal species (Khan, Jo, and Tariq 2015). Pork with higher fat content exhibits significantly higher HAA content compared to lean pork loins (Bassam, Noleto-Dias, and Farag 2022). Lipids play multiple roles in flavor formation during the thermal processing of meat. They serve as solvents for volatile compounds generated during processing, and the thermal oxidation products of lipids can react with components of lean meat tissue, resulting in distinctive flavors (Teng et al. 2023). Additionally, higher moisture content has been associated with increased HAA formation (Alaejos and Afonso 2011). Therefore, the selection of suitable meat matrices and ingredients with lower fat and moisture contents can directly reduce the formation of HAAs at the substrate level.

#### 7.2.2 Optimization of processing methods

The methods and conditions used in meat processing play a critical role in both flavor development and the formation of HAAs, as most volatile compounds are generated through thermally induced reactions (Meurillon and Engel 2016). Cooking temperature, time, pH, and moisture content are particularly important factors influencing the formation of pyrazine in thermally processed meat (Bailey et al. 1994). Cooking temperature and time have a significant impact on both HAA formation in meat products and the sensory properties of cooked food, with the HAA content generally increasing with higher values of these parameters (Dong et al. 2020). The cooking temperature primarily affects the formation of flavor compounds through the Maillard reaction and lipid oxidation processes (Teng et al. 2023). During cooking, sulfur-containing compounds in beef undergo continuous reduction, which is closely associated with nitrogen-containing compounds such as pyrazine and thiazole, leading to changes in HAA production and flavor characteristics (Yu, Cui, and Zhang 2020). The optimization of processing methods has been shown to effectively inhibit HAA formation. However, the impact of these methods on flavor compounds remains unclear.

#### 7.2.3 Addition of exogenous inhibitors

In contrast, there is significant research interest in utilizing exogenous inhibitors to suppress the formation of HAAs. The addition of various polyphenols, such as those derived from peppers, piper fruits, pomegranates, grape seeds, tomatoes, hawthorn, and other fruits, has been shown to reduce the formation of HAAs in meat products (Meurillon and Engel 2016). Additionally, numerous studies have explored the effects of different species on HAA formation dependent on the content of flavonoids and polyphenols (Teng et al. 2023). The use of polyphenols as inhibitors of HAA formation also affects the development of flavor. First, polyphenols can interact with dicarbonyl compounds, which serve as precursors for various flavor compounds, such as pyrazine, pyridine, furanone, thiazole, and thiophene. By acting as scavengers of dicarbonyl compounds, polyphenols inhibit the formation of flavor compounds through the Maillard reaction. Furthermore, polyphenols or their degradation products may react with existing flavor compounds. For example, degradation products of ferulic acid can react with pyrazine, which is crucial not only for HAA formation but also for flavor development (Chen, Cui, and Feng 2022). Cooking with natural antioxidants rich in thiol compounds, such as onions and garlic, can also effectively reduce the formation of HAAs in meat products (Janoszka 2010). The antioxidant activity of fruit extracts has been found to impact Amadori rearrangement during the early stage of the Maillard reaction, leading to a decrease in HAA formation in grilled fish, accompanied by flavor changes (Li et al. 2022). However, the impact of the interaction between polyphenols and Maillard reaction intermediates, such as Amadori compounds or Heyns compounds, on flavor has not been extensively studied. Several investigations have indicated that polyphenols can interact with Amadori compounds, which are important precursors for the formation of flavor compounds in the Maillard reaction (Yu et al., 2020, Chen et al., 2022). Therefore, the interaction between polyphenols and Amadori compounds is also likely to influence flavor development.

## 8 Trends in research

Since numerous challenges related to HAAs in meat products still require prompt investigation and resolution, future research in this field should prioritize the following areas:

1- Clarifying the critical processes and mechanisms of HAA formation in thermally processed meat products: A comprehensive exploration of meat processing technology can shed light on the key processes involved in HAA formation. This understanding will provide a foundation for future research aimed at implementing various control and inhibition strategies.

2- Determining the processing suitability of different raw materials: It is important to identify suitable raw materials for processing and establish quality standards for various thermally processed meats. Managing and reducing the HAA content in products at the source will be crucial.

3- Optimizing and innovating processing technologies: The complex and diverse heat treatment processes involved in meat product manufacturing can contribute to the synthesis of HAAs. Investigating mechanisms for optimizing and innovating processing technologies to enhance product efficiency represents a practical challenge.

4- Studying targeted inhibition technologies for HAAs in thermally processed meat and elucidating the inhibition mechanisms: Developing techniques that specifically inhibit HAA production while preserving meat quality is an important focus for future research.

## 9 Conclusion and future perspectives

The production of HAAs poses a safety risk during the processing of meat products. However, there remain several unresolved issues regarding HAAs that require further investigation. Currently, investigation into the formation mechanism of heterocyclic amines (HAAs) remains limited in scope. Given the intricate biochemical reactions taking place in meat substrates, it is crucial to delve deeper into the interplay between the formation of HAAs and the formation of other essential constituents, thus requiring further exploration. Although there is no conclusive evidence linking excessive HAA consumption to an increased risk of cancer in humans, their potential mutagenicity, carcinogenicity, and widespread presence in food have made them the subject of extensive research in various fields. Hence, the extraction, analysis, and monitoring of HAAs from diverse meat sources are imperative. Considering the widespread presence of HAAs, it is difficult for individuals to completely eliminate their consumption, necessitating considerable endeavors to mitigate the risks posed to human health. Consequently, the exploration of novel natural antioxidants has emerged as a prominent area of research. However, researchers are still far from attaining a comprehensive understanding of all pertinent aspects concerning HAAs.

## Declaration of Competing Interest

The authors declare that they have no known competing financial interests or personal relationships that could have appeared to influence the work reported in this paper.

## Data Availability

No data was used for the research described in the article.
