# Supplementary material for: Unveiling heterocyclic aromatic amines (HAAs) in thermally processed meat products: Formation, toxicity, and strategies for reduction – A comprehensive review
Source: Food Chem X. 2023 Aug 9;19:100833. doi: 10.1016/j.fochx.2023.100833 (PMC10534170; doi:10.1016/j.fochx.2023.100833)
Supplement: Supplementary data 1 [file mmc1.docx]

Table S1. Comparison of key content and highlights between various reviews on HAAs.

| **Title** | **Contents** | **Highlights** | **References** |
| --- | --- | --- | --- |
| Recent advances in heterocyclic aromatic amines: An update on food safety and hazardous control from food processing to dietary intake. | 1. Formation of HAAs (AαC, IQ-type HAA, PhIP, Harman and Norharman) 2. Occurrence and general recognition of HAAs 3. Analytical techniques of HAAs (Sample preparation, analytical methods used to measure HAAs) 4. Impact factors for the formation of HAAs (Cooking temperature and time, precursors, contents, kinetics of HAA formation) 5. Ways to mitigating HAAs in meat and processed meat (Modification of thermal processing conditions, the use of additives) 6. Role of enzymes in the metabolism of HAAs (HAA metabolism, role of P450s, peroxidases, NATs, SULTs, UGTs, glutathione S-transferases) 7. Biomarkers of exposure to HAAs (Urinary biomarkers, PhIP hair levels, DNA adducts, hemoglobin adducts, serum albumin adducts) 8. Risk assessment of HAAs and related “red meat” (mutagenesis and carcinogenesis of HAAs, risk assessment) | - Role of enzymes in the metabolism of HAA; - Biomarkers of exposure to HAAs. | (Chen et al. 2020) |
| Potential carcinogenic heterocyclic aromatic amines (HAAs) in foodstuffs: Formation, extraction, analytical methods, and mitigation strategies. | 1. Formation of HAAs (IQ or IQx type HAAs, IP type HAAs, pyrolytic HAAs) 2. Extraction and clean-up methods for HAAs (Solid-phase extraction, solid-phase microextraction, dispersive liquid–liquid microextraction, other extraction and clean-up methods) 3. Instrumental analytical methods (GC‒MS, LC based methods, LC‒MS or LC‒MS/MS, Other analytical methods) 4. Mitigation strategies for HAAs (Adjusting cooking methods or process conditions; addition of natural product extracts, antioxidants, or other compounds; reasonable selection of foodstuff types) | - Mitigation strategies for HAAs (Adjusting cooking methods or process conditions; addition of natural product extracts, antioxidants, or other compounds; reasonable selection of foodstuff types) | (Dong et al. 2020) |
| Heterocyclic aromatic amines in meat: Formation, isolation, risk assessment, and inhibitory effect of plant extracts. | 1. Occurrence of HAAs 2. HAA formation 3. Types of HAAs 4. Quantification and Identification 5. HAA risk assessment 6. HAA inhibition by Natural extracts 7. Suggested mechanism for an inhibitory effect to decrease potential carcinogenic constituents (The action of antioxidative products; inhibition of Maillard reaction) | - Suggested mechanism for an inhibitory effect to decrease potential carcinogenic constituents (The action of antioxidative products; inhibition of Maillard reaction) | (Nadeem et al. 2021) |
| Consumption of thermally processed meat containing carcinogenic compounds (polycyclic aromatic hydrocarbons and heterocyclic aromatic amines) versus a risk of some cancers in humans and the possibility of reducing their formation by natural food additives—a literature review | 1. Polycyclic aromatic hydrocarbons (PAHs) in food 2. Heterocyclic aromatic amines (HAAs) in food 3. Mechanisms of bioactivation (PAHs, HAAs) 4. Risk of cancer (Head and Neck Cancer; Gastrointestinal Cancer: Esophageal Cancer, Pancreatic Cancer, Gastric Cancer, Colorectal Cancer; Prostate Cancer; Lymphatic Cancer; Renal and Bladder Cancer; Breast Cancer) 5. Reducing the risk of cancer by supplementing the diet with plant products 6. Influence of vegetable additives on the synthesis of carcinogenic PAHs and HAAs in thermally processed meat dishes | - Risk of various cancer | (Bulanda and Janoszka 2022) |
| Unveiling Heterocyclic Aromatic Amines (HAAs) in Protein-Rich Foods: Formation, Toxicity, and Strategies for Reduction - A Comprehensive Review | 1. Formation mechanisms of HAAs (AIAs, ACs) 2. Distribution of heterocyclic aromatic amines (HAAs) in thermally processed meat 3. Toxicity of HAAs (Carcinogenesis, Mutagenicity, Risk assessment) 4. Detection methods of HAAs (Pretreatment, Detection) 5. Inhibition mechanism of HAAs (controlling precursors; regulating intermediates: scavenging free radicals and controlling reactive carbonyl species; promoting metabolism.) 6. The Effect of inhibiting the formation of HAAs on flavor substances   The formation of flavor substances in thermally processed meat  The effect of different inhibition strategies on the formation of HAAs and flavor substances in thermally processed meat (Rational selection of food types; Optimization of processing methods; Addition of exogenous inhibitors)   1. Trends in research | - Distribution of heterocyclic aromatic amines (HAAs) in thermally processed meat; - Inhibition mechanism of HAAs (controlling precursors; regulating intermediates: scavenging free radicals and controlling reactive carbonyl species; promoting metabolism); - The Effect of inhibiting the formation of HAAs on flavor substances |  |

**References:**

Bulanda, Sylwia, and Beata Janoszka. 2022. "Consumption of thermally processed meat containing carcinogenic compounds (polycyclic aromatic hydrocarbons and heterocyclic aromatic amines) versus a risk of some cancers in humans and the possibility of reducing their formation by natural food additives—a literature review." *Int J Env Res Pub He* 8 (19):4781. doi: 10.3390/ijerph19084781.

Chen, X., W. Jia, L. Zhu, L. Mao, and Y. Zhang. 2020. "Recent advances in heterocyclic aromatic amines: An update on food safety and hazardous control from food processing to dietary intake." *Compr Rev Food Sci Food Saf* 19 (1):124-148. doi: 10.1111/1541-4337.12511.

Dong, H., Y. Xian, H. Li, W. Bai, and X. Zeng. 2020. "Potential carcinogenic heterocyclic aromatic amines (HAAs) in foodstuffs: Formation, extraction, analytical methods, and mitigation strategies." *Compr Rev Food Sci Food Saf* 19 (2):365-404. doi: 10.1111/1541-4337.12527.

Nadeem, H. R., S. Akhtar, T. Ismail, P. Sestili, J. M. Lorenzo, Mman Ranjha, L. Jooste, C. Hano, and R. M. Aadil. 2021. "Heterocyclic Aromatic Amines in Meat: Formation, Isolation, Risk Assessment, and Inhibitory Effect of Plant Extracts." *Foods* 10 (7). doi: 10.3390/foods10071466.
